# The cancer-associated *SF3B1*^K700E^ spliceosome mutation confers enhanced sensitivity to BV-6-induced cytotoxicity

**DOI:** 10.1038/s41419-025-07790-y

**Published:** 2025-07-01

**Authors:** Lydia E. Roets, Jaine K. Blayney, Hayley P. McMillan, Patrick J. Preston, Alexander M. Mutch, Ken I. Mills, Kienan I. Savage, Katrina M. Lappin

**Affiliations:** Patrick G Johnston Centre for Cancer Research, Queen’s University Belfast, Belfast, UK

**Keywords:** Apoptosis, Cancer

## Abstract

Recurrent somatic mutations in the key spliceosome component, *SF3B1*, have been identified at various frequencies across several cancer types. The most common hotspot mutation is the K700E missense mutation, and while its effects on splicing have been well characterised at the molecular level, the mis-spliced genes that contribute to cancer progression and/or dictate responses to therapy are still unclear. Here, we used we use cell line modelling to assess the impact of the SF3B1^K700E^ mutation on the cellular response to various apoptosis-inducing agents. Our data suggest that the SF3B1^K700E^ mutation leads to reduced cFLIP levels, along with defects in the splicing and translation of *BCL2*, causing a shift in the balance of pro- and anti-apoptotic genes and proteins, which confers greater sensitivity to the bivalent SMAC mimetic, BV-6. As such, BV-6 may represent a therapeutic opportunity for patients with *SF3B1* mutant cancers.

## Introduction

The spliceosome is a multi-megadalton ribonucleoprotein complex that catalyses nuclear RNA processing and maturation [1]. This involves the removal of intronic sequences from precursor messenger RNA (mRNA), followed by the ligation of remaining exons to form mature protein-coding mRNA transcripts. Given the crucial role of the splicing machinery in the processing of mRNA transcripts, most of the genes encoding components of the spliceosome are intolerant to loss-of-function mutations [2]. Instead, many human cancers exhibit abnormal splicing patterns due to the altered functionality of mutated splicing factors.

SF3B1 (splicing factor 3b subunit 1), which aids in the recognition of consensus sequences in introns to promote efficient mRNA splicing, is the most frequently mutated splicing factor in cancer, with particular enrichment in various haematological malignancies—including acute myeloid leukaemia [3], myelodysplastic syndromes and chronic lymphocytic leukaemia [4], as well as uveal melanomas [5, 6] and other solid tumours such as skin [7] and breast cancers [8]. *SF3B1* mutations are almost exclusively missense mutations that alter the ability of SF3B1 to recognise 3’ splice sites, often leading to cryptic splice site usage that can result in disruption of target gene open reading frames. Approximately half of the aberrant transcripts produced are subject to nonsense-mediated decay (NMD), resulting in reduced transcript levels and protein expression [9].

While the connection between *SF3B1* mutations and their effects on splicing has been well-characterised at the molecular level [10], the precise ways in which the dysregulated gene expression influences carcinogenesis or affects response to treatment remain unclear. Nonetheless, the ability to therapeutically target *SF3B1* mutant cells would be clinically useful for the treatment of multiple cancer types. To address this, we utilised our previously generated isogenic SF3B1^K700E^ cell line model [11], and screened for apoptosis-inducing compounds with selective activity in SF3B1^K700E^ cells, identifying the bivalent SMAC mimetic, BV-6. Our data suggest that the SF3B1^K700E^ mutation causes a subtle shift in the balance of pro- and anti-apoptotic transcripts and proteins—particularly cFLIP and BCL-2, resulting in apoptotic priming and resulting in increased sensitivity to BV-6. As such, BV-6 may represent a therapeutic opportunity for patients with *SF3B1* mutant cancers.

## Results

### The cancer-associated SF3B1^K700E^ mutation confers enhanced sensitivity to the SMAC mimetic BV-6

**Fig. 1:**
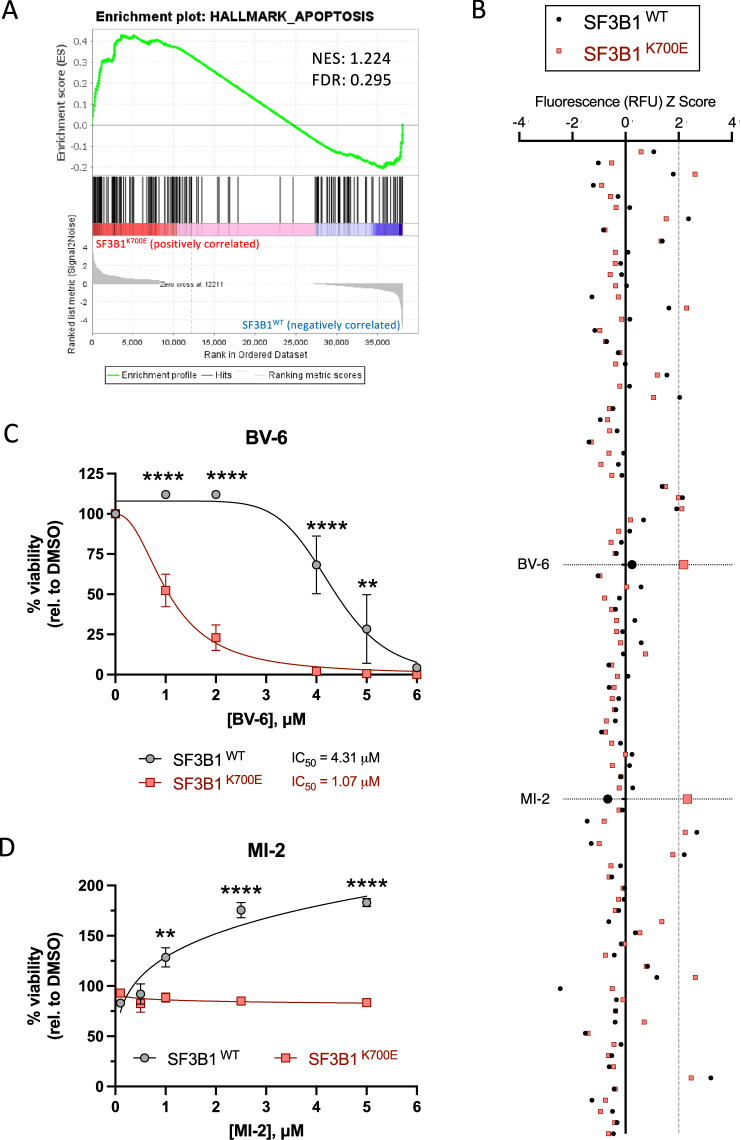
The cancer-associated SF3B1 missense mutation, K700E, confers enhanced sensitivity to the SMAC mimetic, BV-6. **A** Gene set enrichment analysis (GSEA) plot depicting enrichment of the Apoptosis Hallmark SF3B1^K700E^ cells (*n* = 3). NES normalised enrichment score, FDR false discovery rate. **B** *Z*-scores plotted for each of the 89 apoptosis-inducing agents screened against our SF3B1^WT^ (red) and SF3B1^K700E^ (black) cells. Dose-responses following a 72 h incubation with (**C**) BV-6 in SF3B1 wild-type (SF3B1^WT^) and mutant (SF3B1^K700E^) K562 cells. (Mean ± SEM; *n* = 3; 2-way ANOVA with Šidák’s multiple comparisons) and (**D**) MI-2 (Mean ± SEM; *n* = 2; 2-way ANOVA with Šidák’s multiple comparisons). ^*^P < 0.05; ^**^P < 0.01; ^***^P < 0.001; ^****^P < 0.0001.

*SF3B1* mutations change how the spliceosome component recognises 3’ splice sites, resulting in cryptic splice site usage. This often leads to exon skipping or intron retention that can interfere with the open-reading-frame of target genes, culminating in disrupted gene expression [9, 11]. With this in mind, we performed GSEA on normalised gene counts derived from the sequencing of total RNA extracted from SF3B1^WT^ and SF3B1^K700E^ K-562 cells. GSEA identified ‘Apoptosis’ as an enriched pathway/hallmark associated with the SF3B1^K700E^ mutation (Fig. 1A). Consequently, sensitivity to 89 apoptosis-inducing agents (Supplementary Table 1) was assessed in our isogenic K-562 cell model to investigate whether the SF3B1^K700E^ mutation conferred any therapeutic vulnerabilities. Cell viability was assessed using CellTox Green after 72 h with *Z*-score analysis identifying two compounds of interest, BV-6 and MI-2, which preferentially targeted SF3B1^K700E^ cells (Fig. 1B). Validation of the two “hits” was carried out with CellTiter-Glo (Fig. 1C, D).

BV-6 is a synthetic small-molecule peptide that mimics second mitochondria-derived activator of caspases (SMAC) or direct inhibitor of apoptosis (IAP)-binding protein with low pI (DIABLO), as it is otherwise known. SMAC is a mitochondrial protein that is released into the cytosol where it binds to IAPs and thereby shifts the cells' homeostatic balance in favour of caspase activation and programmed cell death [12]. In line with our screening data (Fig. 1B), SF3B1^K700E^ cells proved to be significantly more sensitive to the bivalent SMAC mimetic (SM), BV-6, when compared to their WT counterparts (Figs. 1C and S1A, C). To determine whether the observed SF3B1^K700E^-mediated sensitivity was specific to BV-6, we tested another bivalent SM–Birinapant in our isogenic model, which also exerted SF3B1^K700E^-specific cytotoxic effects (Fig. S1B, D). This suggests that the SF3B1^K700E^ mutation primes cells for SM-induced cell death.

Similarly, a lung mesothelioma-derived cell line (H-2595), a pancreatic cancer cell line (Panc05.04) and an isogenic ALL model (Nalm-6), all harbouring the SF3B1^K700E^ mutation also exhibited enhanced sensitivity to BV-6 when compared to matched SF3B1^WT^ lines (Fig. S1E–G), demonstrating that SF3B1^K700E^-mediated sensitivity to BV-6 is not cell-line-specific.

MI-2 induces specific chromatin changes to disrupt the interaction between menin and MLL1, thereby blocking MLL fusion protein-mediated leukaemic transformation. In contrast to BV-6 (Fig. 1C), MI-2 exerted cytostatic effects on the SF3B1^K700E^ cells while enhancing proliferation in the SF3B1^WT^ cells (Fig. 1D). Consequently, only BV-6 was further investigated.

To confirm that BV-6 inhibits both cellular IAP (cIAP) and X-linked IAP (XIAP), expression levels of these proteins were assessed following treatment with BV-6 (Fig. S1H). As expected, cIAP1 and XIAP were degraded in a dose-dependent manner, and more so in the K700E cells (Fig. S1I, J). This is unsurprising given that cIAPs and XIAP are constitutively organised and stabilised in heteromeric complexes [13]. cIAP2 was undetectable in our K-562 cells with two independent antibodies (data not shown).

### BV-6-induced cytotoxicity is dependent on autocrine TNFα production

**Fig. 2:**
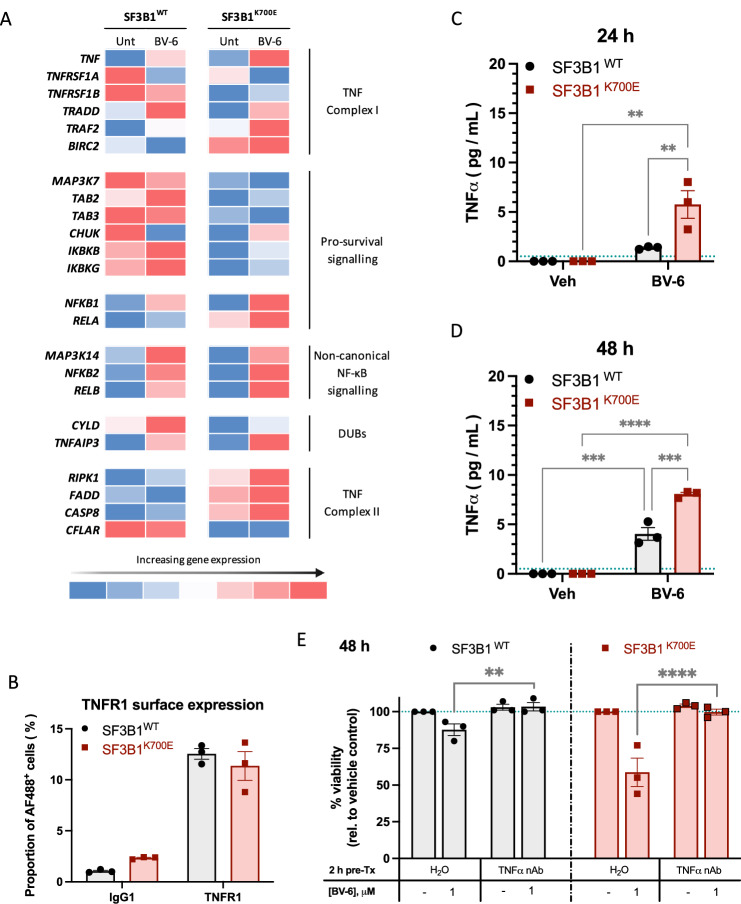
BV-6-induced cytotoxicity is dependent on autocrine TNFα production. **A** Heatmap of the mean normalised counts of selected genes from within the TNF-α signalling and NF-κB pathways for SF3B1^WT^ and SF3B1^K700E^ cells at baseline and following a 48 h incubation with 1 μM BV-6 (*n* = 3). **B** Characterisation of TNFR1 surface expression for *SF3B1*^WT^ and *SF3B1*^K700E^ cell lines, represented as the percentage of AF488-positive cells, with non-specific signal ascertained using an IgG1 isotype control. **C**, **D** Autocrine (soluble) TNFα levels within the cell culture media of SF3B1^WT^ and SF3B1^K700E^ cells, following (**C**) 24- and (**D**) 48-h incubations with 0.1% DMSO vehicle or 1 μM BV-6 (Mean ± SEM; *n* = 3; 2-way ANOVA with Tukey’s multiple comparisons). **E** Viability assessment of *SF3B1*^WT^ and *SF3B1*^K700E^ cells pre-treated with vehicle alone or 1 μg/mL TNFα neutralising antibody, followed by a 48 h incubation with 1 μM BV-6 (Mean ± SEM; *n* = 3; 2-way ANOVA with Šidák’s multiple comparisons). *P < 0.05; **P < 0.01; ***P < 0.001; ****P < 0.0001.

To help ascertain the mechanism of BV-6-induced cell death, the transcriptomic profiles of baseline and BV-6-treated SF3B1^WT^ and SF3B1^K700E^ cells were assessed. GSEA identified TNF-α signalling via NF-κB to be of interest, with positive enrichment of the pathway in BV-6-treated SF3B1^K700E^ cells (Fig. S2A). In line with this, BV-6 treatment upregulated *TNF*, *NFKB1*, *RELA*, *NFKB2*, and *RELB*, with greater impact in the SF3B1^K700E^ cells compared to SF3B1^WT^ cells (Fig. 2A). Fractionated Western blot analysis of NF-κB1 and NF-κB2 proteins provided orthogonal validation of upregulated NF-κB signalling and shows greater contribution of the non-canonical NF-κB signalling pathway in SF3B1^K700E^ cells (Fig. S2B), with clear translocation of RelB into the nucleus of our SF3B1 mutant model. To determine whether altered TNFR1 surface expression could account for enrichment of the TNF-α signalling pathway, TNFR1 protein levels were assessed via flow cytometry but were not significantly altered in SF3B1^K700E^ cells (Figs. 2B and S3A, B).

Typically, cells can be grouped into three general classes based on their response to SMs. Class-I cells upregulate autocrine TNF-α production and cell death following exposure to SMs [14–16]. Class-II cells exhibit increased autocrine TNF-α production in response to SM treatment, but the cell population is otherwise largely unaffected, and in class-III cells, SMs have no effect on autocrine TNF-α production or on cell death [17]. Furthermore, it has been proposed that the extent of autocrine TNF-α induction and the relative levels of pro- and anti-apoptotic proteins may distinguish class-I from class-II cells [17]. Based on this, we assessed secreted TNF-α levels in the media of cells treated with BV-6 and observed significant dose-dependent upregulation of autocrine TNF-α in the K562 and pancreatic SF3B1^K700E^ models, following BV-6 treatment (Figs. 2C, D and S3C). Interestingly, while the NALM-6 SF3B1^K700E^ model experienced significantly more TNFα secretion when untreated, both these cells and the H2595 SF3B1^K700E^ line, did not experience any dose-dependent increase in TNFα secretion upon BV-6 treatment (Fig. S3C). This suggests that these cells may be more reliant on another death receptor ligand (TRAIL or FasL) to induce programmed cell death.

To provide conclusive evidence for whether TNF-α is required by our K562 and pancreatic SF3B1^K700E^ models for BV-6-induced cytotoxicity, cells were pre-treated with a TNF-α-neutralising antibody prior to BV-6 treatment. This resulted in significant rescue of BV-6-induced cell death, which is particularly evident in the SF3B1^K700E^ cells (Fig. 2E). Taken together, these data indicate that BV-6-induced cytotoxicity involves the interplay of the TNF superfamily death receptor ligands, with greater sensitivity always observed in the SF3B1^K700E^ models.

### BV-6 induces programmed cell death via a RIP(K1)-dependent extrinsic apoptotic pathway

**Fig. 3:**
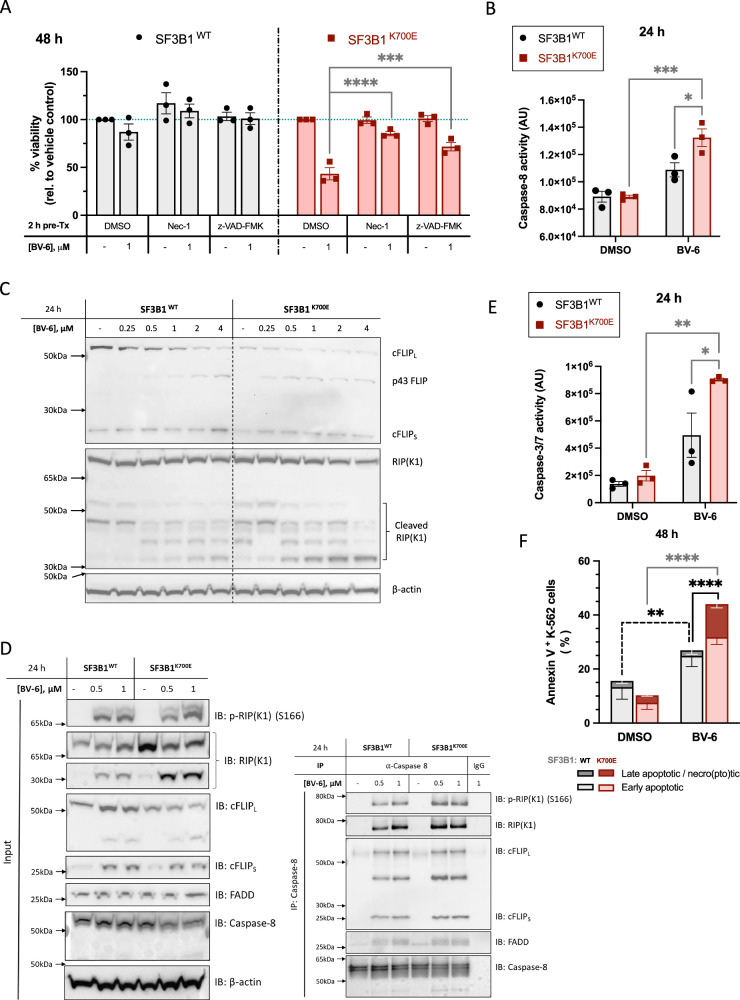
BV-6 induces programmed cell death via a RIP(K1)-dependent apoptotic pathway. **A** Viability assessment of SF3B1^WT^ and SF3B1^K700E^ cells pre-treated for 2 h with DMSO vehicle control, 10 μM necrostatin-1, or 10 μM z-VAD-FMK, followed by a 48 h incubation with 0.1% DMSO vehicle or 1 μM BV-6 (Mean ± SEM; *n* = 3; 2-way ANOVA with Dunnett’s multiple comparisons). **B** Caspase-8 activity following 24 h treatment with 0.1% DMSO vehicle or 1 μM BV-6 in SF3B1^WT^ and SF3B1^K700E^ cells (Mean ± SEM; *n* = 3; 2-way ANOVA with Tukey’s multiple comparisons). **C** Representative Western blot analysis of cFLIP and RIP(K1) protein expression in SF3B1^WT^ and SF3B1^K700E^ cells following treatment with the indicated concentrations of BV-6 for 24 h. **D** Co-immunoprecipitation assay of RIP(K1), pro-caspase-8, cFLIP and FADD following immunoprecipitation of caspase-8 from SF3B1^WT^ and SF3B1^K700E^ cells treated with the indicated concentrations of BV-6 for 24 h in the presence of 20 μM z-VAD-FMK. **E** Activity levels of caspases-3/7 in SF3B1^WT^ and SF3B1^K700E^ cells following BV-6 treatment (Mean ± SEM; *n* = 3; 2-way ANOVA with Tukey’s multiple comparisons). **F** Proportion of Annexin V^+^ cells following a 48 h incubation with the indicated concentrations of BV-6 in SF3B1^WT^ and SF3B1^K700E^ cells (Mean ± SEM; *n* = 4; 2-way ANOVA with Tukey’s multiple comparisons to compare the total proportions of apoptotic cells, i.e., combined Annexin V^+^/7-AAD^+^ and Annexin V^+^/7-AAD^−^ cells). ^*^P < 0.05; ^**^P < 0.01; ^***^P < 0.001; ^****^P < 0.0001.

Pharmacological inhibitors of programmed cell death were utilised to establish the mechanism(s) underlying BV-6-induced cytotoxicity. Given the association of *SF3B1* mutations with abnormal splicing of genes involved in iron metabolism in haematopoietic cells [18, 19], we pre-treated the cells with ferrostatin-1 but found no significant contribution of ferroptosis to BV-6-induced cell death (Fig. S4A). However, pre-treatments with necrostatin-1, which blocks the kinase activity of RIP(K1), and the pan-caspase inhibitor, z-VAD-FMK, both conferred significant protection against BV-6-induced cell death, particularly in SF3B1^K700E^ cells (Fig. 3A). In contrast, pre-treatment with another necroptosis inhibitor, necrosulfonamide, which blocks the Mixed Lineage Kinase domain-Like (MLKL) protein, was unable to rescue cell viability (Fig. S4A). To examine this further, we blotted for components of the necro(pto)some complex but could not detect the presence of RIPK3 (data not shown), which is essential for necroptosis [20]. Combined, this suggests an inability of K-562 cells to undergo necroptosis, and instead points towards the induction of RIP(K1) kinase activity-dependent apoptosis by BV-6.

As caspase-8 is one of the initiator caspases associated with the extrinsic apoptotic pathway, we next assessed caspase-8 activity within both SF3B1^WT^ and SF3B1^K700E^ cells following BV-6 treatment, which showed significantly more caspase-8 activity in the SF3B1^K700E^ cells compared to the WT control (Figs. 3B and S4B). Additionally, we assessed the cleavage of two target substrates of caspase-8: (1) the endogenous caspase-8 inhibitor and suppressor of extrinsic apoptosis, cFLIP^21^; and (2) RIP(K1)^22^. Indeed, proteolytic cleavage of the long isoform of cFLIP (cFLIP_L_) and RIP(K1) appeared to increase in a BV-6 dose-dependent manner (Fig. 3C). Importantly, we observed reduced expression of cFLIP in SF3B1^K700E^ cells, as well as increased BV-6-induced cleavage of cFLIP_L_ in these cells compared to their SF3B1^WT^ counterparts (Fig. 3C). Furthermore, we assessed the formation of the TNF complex II in SF3B1^WT^ and SF3B1^K700E^ cells following BV-6 treatment using caspase 8 immunoprecipitation assays. Whilst not a quantitative method, this revealed substantial formation of the cell death-inducing signalling complex in SF3B1^K700E^ cells at 0.5 µM BV-6 treatment, in comparison to SF3B1^WT^ cells (Figs. 3D and S4C). In contrast, there is slightly less TNF complex II precipitated from SF3B1^K700E^ cells treated with 1 µM BV-6 compared to with SF3B1WT counterparts. This is likely due to a combination of increased SF3B1^K700E^ cell death (evidenced by increased CASP8 cleavage) and complex II formation saturation with this dose of BV6. In line with this, activity of the executioner caspases-3/7 was significantly enhanced in SF3B1^K700E^ cells compared to SF3B1^WT^ cells following BV-6 treatment (Figs. 3E and S4B). Moreover, the proportions of apoptotic cells were significantly elevated in SF3B1^K700E^ cells compared to their WT counterparts (Figs. 3F and S5A, B).

### SF3B1^K700E^ cells contain reduced levels of cFLIP, predisposing them to BV-6-induced cytotoxicity

**Fig. 4:**
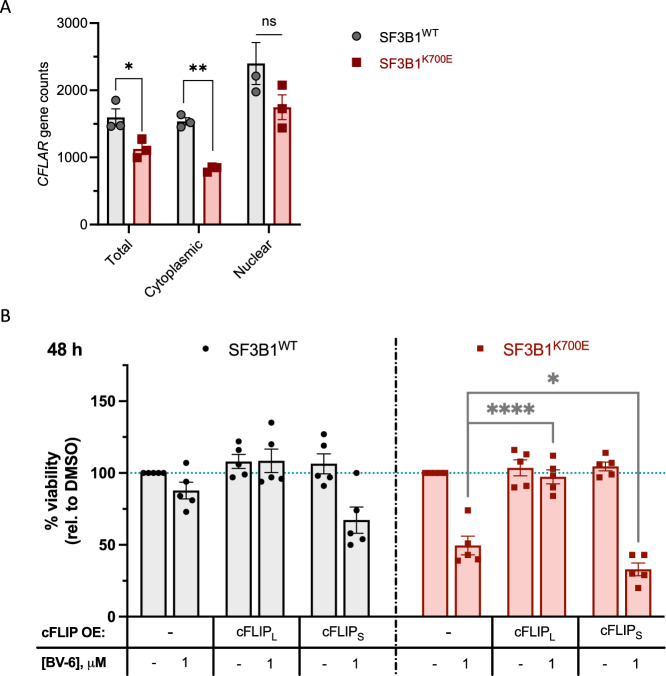
SF3B1^K700E^-mutated cells contain significantly reduced cFLIP protein levels, which predispose the cells to BV-6-induced cytotoxicity. **A** Normalised total, nuclear and cytoplasmic *CFLAR* gene counts from SF3B1^WT^ and SF3B1^K700E^ cells (Mean ± SEM; *n* = 3; Unpaired *t*-test). **B** Viability assessment of parental and cFLIP_L_- or cFLIP_S_-overexpressing SF3B1^WT^ and SF3B1^K700E^ cells treated with BV-6 for 48 h (Mean ± SEM; *n* = 5; 2-way ANOVA with Dunnett’s multiple comparisons). ^*^P < 0.05; ^**^P < 0.01; ^***^P < 0.001; ^****^P < 0.0001.

As previously mentioned, reduced cFLIP protein expression was observed in the SF3B1^K700E^ cells when compared with their WT counterparts (Fig. 3C). Given the role of SF3B1 in pre-mRNA splicing, we examined the transcription and splicing of the cFLIP encoding gene *CFLAR*. Indeed, normalised gene counts derived from total RNA demonstrate downregulation of *CFLAR* expression in SF3B1^K700E^ cells compared to their WT counterparts (Fig. 4A). Additionally, we have previously shown that *SF3B1* mutation-driven splicing defects can result in impaired nuclear-to-cytoplasmic export of mis-spliced transcripts [11]. In line with this, *CFLAR* transcripts were significantly downregulated in the cytoplasmic fraction of SF3B1^K700E^ cells compared to their SF3B1 wild-type counterparts (Fig. 4A). Moreover, analysis of RNA sequencing (RNA-seq) data following treatment with an SMG1 inhibitor to inhibit NMD highlighted the inclusion of cryptic splice sites at the 3’ end of exon 5 and the 5’ end of exon 6 within the *CFLAR* transcripts. These transcripts are usually degraded by NMD (Fig. S6A), which likely accounts for the reduced *CFLAR* transcript in the cytoplasm and subsequent depletion of cFLIP protein expression in SF3B1^K700E^ cells at baseline. To validate this finding, we designed custom primers to detect the inclusion of intronic sequences, which create the cryptic splice sites at exons 5 and 6. This primer set is only able to detect an amplicon in the SF3B1^K700E^ mutant line following treatment with SMG1 inhibitor (Fig. S6B). Usage of this cryptic splice site was detected in all SF3B1 mutant cell lines tested (Fig. S6B).

In line with this, the H-2595 lung mesothelioma cells, carrying the SF3B1^K700E^ mutation, expressed significantly lower levels of the *CFLAR* transcript and cFLIP protein compared to matched SF3B1^WT^ H-2591 cells (Fig. S6C, D). Combined, these data suggest that SF3B1^K700E^ causes altered splicing of the *CFLAR* gene, leading to NMD, resulting in reduced cFLIP_L_ and cFLIP_S_ protein expression.

To investigate whether this reduced cFLIP expression contributed to the sensitivity of SF3B1^K700E^ cells to BV-6, we stably expressed cFLIP_L_ or cFLIP_S_ transgenes in our isogenic K-562 cell lines (Fig. S6E). Surprisingly, whilst ectopic expression of cFLIP_L_ rescued the modest induction of cell death observed in the SF3B1^WT^ cells, it also completely rescued SF3B1^K700E^ cell sensitivity to BV-6 (Fig. 4B). In contrast, ectopic expression of cFLIP_S_ failed to protect either cell line from BV-6-mediated cell death (Fig. 4B).

### *SF3B1*^*K700E*^ cells exhibit reduced BCL-2, promoting the intrinsic apoptotic pathway and further predisposing the cells to BV-6-induced cytotoxicity

**Fig. 5:**
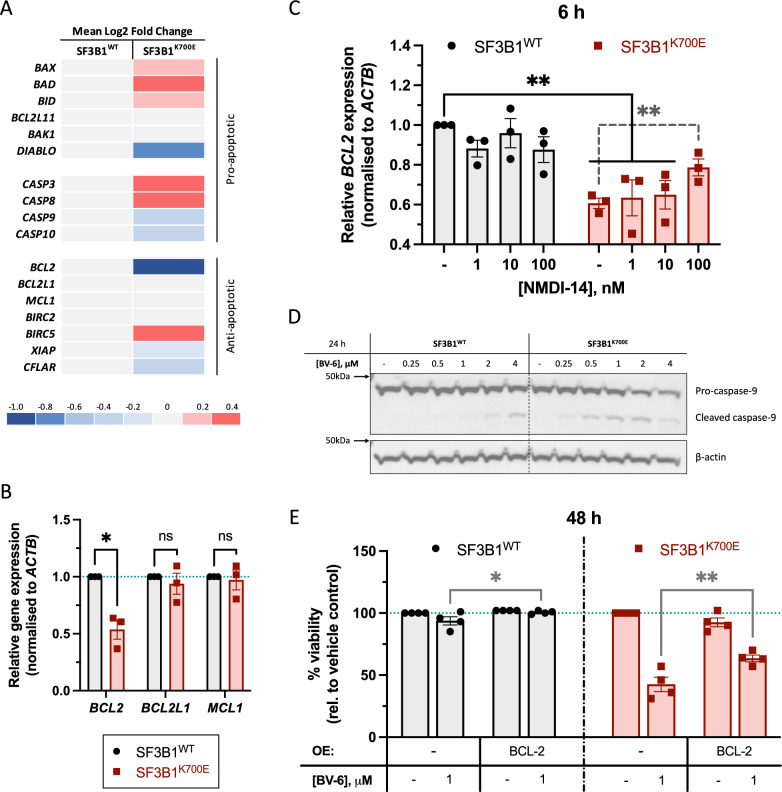
SF3B1^K700E^ mutated K-562 cells exhibit aberrant splicing of *BCL2* resulting in reduced transcript and protein levels, predisposing the cells towards BV-6-induced cytotoxicity. **A** Heatmap highlighting the mean log2 fold change of various pro- and anti-apoptotic genes in SF3B1^WT^ and SF3B1^K700E^ cells (*n* = 3). **B** qRT-PCR validation of the transcriptomic data for *BCL2*, *BCL2L1*, and *MCL1* genes in SF3B1^WT^ and SF3B1^K700E^ cells at baseline (Mean ± SEM; *n* = 3; Multiple unpaired *t*-tests with Welch’s correction). **C** Evaluation of *BCL2* gene transcripts in SF3B1^WT^ and SF3B1^K700E^ cells following a 6 h incubation with the indicated concentrations of NMDI-14 (Mean ± SEM; *n* = 3; 2-way ANOVA with Dunnett’s multiple comparisons; Paired *t*-test). **D** Representative Western blot showing caspase-9 cleavage in SF3B1^WT^ and SF3B1^K700E^ cells following treatment with the indicated concentrations of BV-6 for 24 h. **E** Viability assessment of parental SF3B1^WT^ and SF3B1^K700E^ cells and cells stably overexpressing *BCL2* following a 48 h treatment with BV-6 (Mean ± SEM; *n* = 4; 2-way ANOVA with Dunnett’s multiple comparisons). ^*^P < 0.05; ^**^P < 0.01; ^***^P < 0.001; ^****^P < 0.0001.

In line with the GSEA data (Fig. 1A), the striking downregulation of *BCL2* along with partial upregulation of pro-apoptotic *BAX*, *BAD*, and *BID* in SF3B1^K700E^ cells (Fig. 5A) suggests mutation-induced priming of the K-562 cells towards apoptosis. Additionally, given the concurrent downregulation of *DIABLO* (protein: SMAC) (Fig. 5A), the cytotoxic effects exerted by the bivalent SMs, BV-6 (Figs. 1C and S1A, B) and Birinapant (Fig. S1D, E), on SF3B1^K700E^ cells are unsurprising.

BCL-2 downregulation in SF3B1^K700E^ cells was validated at both the mRNA and protein levels (Figs. 5B and S7A). To investigate the potential involvement of differential splicing in the marked reductions of *BCL2* expression, we used EventPointer to assess our RNA-seq data but were unable to detect any events associated with the *BCL2* gene (Supplementary Table 2). Despite this, we treated SF3B1^WT^ and SF3B1^K700E^ cells with the NMD inhibitor, NMDI-14, and found that the 100 nM dose significantly upregulated *BCL2* expression within SF3B1^K700E^ cells relative to their vehicle control, and partially rescued *BCL2* expression back towards the baseline transcript levels observed in SF3B1^WT^ cells (Fig. 5C). This suggests that rapid degradation of aberrant *BCL2* mRNA via NMD is at least partly responsible for the reduced transcript and protein levels. Moreover, BV-6 dose-dependent cleavage of caspase-9 is enhanced in SF3B1^K700E^ cells (Figs. 5D and S7B), indicating involvement of the intrinsic apoptotic pathway to amplify the overall cytoplasmic apoptotic signalling.

To complement the RNA-seq and qRT-PCR data derived from our isogenic SF3B1 cell model, similar analyses were conducted on CD34^+^-enriched bone marrow samples taken from MDS patients [21] (Fig. S7C) and matched lung mesothelioma cell lines (Fig. S7D), with and without the SF3B1^K700E^ mutation. Notably, and in keeping with the in vitro results (Fig. 5A, B), *BCL2* transcripts were significantly decreased in the patient samples and lung mesothelioma cells with the K700E mutation, compared to those without (Fig. S7C, D). Furthermore, Western blot analysis demonstrated reduced BCL-2 protein levels within H-2595 (SF3B1^K700E^) cells compared to matched WT control cells (Fig. S7E, F), presumably due to SF3B1^K700E^-mediated mis-splicing of *BCL2*.

To determine the impact of SF3B1^K700E^-mediated BCL-2 downregulation on cellular sensitivity to BV-6, we stably expressed a *BCL-2 trans-*gene in our isogenic SF3B1 model (Fig. S7G). Interestingly, overexpression of the *BCL2 trans*-gene partially rescued SF3B1^K700E^ cells from BV-6-induced cytotoxicity (Fig. 5E).

## Discussion

Despite the essential role of the splicing machinery in the processing and maturation of RNA transcripts, recurrent somatic mutations have been identified in various splicing factors across a range of cancers [22]. This implies functional and pathophysiological consequences of spliceosome mutations in carcinogenesis [23, 24]. The SF3B1^K700E^ mutation has been shown to alter the factor’s recognition of canonical 3’-splice sites, often leading to cryptic splicing of target genes, along with loss or qualitative differences in function at the protein level [10]. Despite this, the precise ways in which SF3B1^K700E^ contributes to cancer progression and/or dictates responses to therapy, remain unclear.

Using our previously generated SF3B1^K700E^ isogenic model [11], we assessed the impact of the SF3B1^K700E^ mutation on response to apoptosis-inducing agents. From this, we identified the bivalent SM, BV-6, as a compound of interest. SMs are synthetic compounds that were modelled on the N-terminal IAP-binding motif of the mitochondrial protein SMAC/DIABLO [25] to antagonise IAPs, thereby enabling them to activate and/or modulate programmed cell death pathways. SMs, such as BV-6, trigger the auto-ubiquitination and subsequent proteasomal degradation of the E3 ligase, cIAP1. cIAP1 regulates extrinsic apoptosis and cellular responses to TNFα by ubiquitinating RIP(K1) [26]. The loss of cIAP1 disrupts the addition of ubiquitin chains to RIP(K1) and promotes the formation of the cytosolic TNF-complex-II, leading to programmed cell death [27]. Further processing of RIP(K1) through caspase-8-mediated proteolytic cleavage [28] is required to proceed with the apoptotic programme [29], which is enhanced in the absence of the endogenous caspase-8 regulator, cFLIP_L_ [30]. Interestingly, we have shown reduced protein levels of cFLIP in the SF3B1-mutant setting, along with altered splicing and reduced *CFLAR* transcript levels in the cytoplasm of SF3B1^K700E^ cells. This is consistent with our previous reports demonstrating reduced splicing and nuclear export of a large subset of genes in SF3B1^K700E^ cells [11]. Therefore, we propose that the cFLIP-deficient conditions caused by SF3B1^K700E^ enhance caspase-8 activation, contributing to the significant cytotoxicity observed in SF3B1^K700E^ cells following BV-6 treatment. To validate the role of cFLIP in the enhanced sensitivity of SF3B1-mutant cells to BV-6, we stably overexpressed both the long (cFLIP_L_) and short (cFLIP_s_) isoforms of cFLIP. Interestingly, only ectopic expression of cFLIP_L_ substantially rescued the cell death observed in our mutant SF3B1 model following BV-6 exposure, with ectopic expression of the cFLIP_s_ isoform having no impact on BV-6-induced cytotoxicity in either cell line. This was somewhat surprising as cFLIP_s_ were previously shown to play a role in inhibiting caspase-8 activation and preventing apoptosis [31]. However, further exploration of the literature highlighted a role for cFLIP_s_ in necroptosis through promotion of the ripoptosome [32]. Therefore, we surmise that the inability of cFLIP_S_ to influence cell viability following BV-6 treatment in our isogenic model was due to its role being redundant, as our K-562 SF3B1 mutant cells do not express RIPK3, which is an important component in ripoptosome formation and therefore necroptosis [20].

Hashimoto and colleagues identified that the combined therapeutic inhibition of XIAP and BCL-2 promotes cell death in aggressive AML [33]. XIAP, unlike other IAP family members, is the only caspase inhibitor with the ability to directly inhibit caspases-3/-7 and -9, thereby enabling effective antagonism of both the intrinsic and extrinsic pathways [34]. In agreement with this, we identified downregulation of anti-apoptotic BCL-2 as an additional mechanism contributing to the enhanced sensitivity of the SF3B1-mutant cells to BV-6, which inhibits both cIAP1/2 and XIAP. We have shown that the downregulation of *BCL-2* transcript and protein levels is a result of NMD in SF3B1^K700E^ cells. Unsurprisingly, NMD inhibition did not protect against BV-6-induced cytotoxicity (data not shown), presumably due to the translation of aberrant dysfunctional BCL-2 protein. Considering the influence of the SF3B1^K700E^ mutation on both *CFLAR* (FLIP) and *BCL2* splicing and expression, along with its broader effects on apoptotic gene regulation, the SF3B1^K700E^ variant appears to exert a polygenic impact on apoptosis, ultimately increasing susceptibility to bivalent SMAC mimetics.

**Fig. 6:**
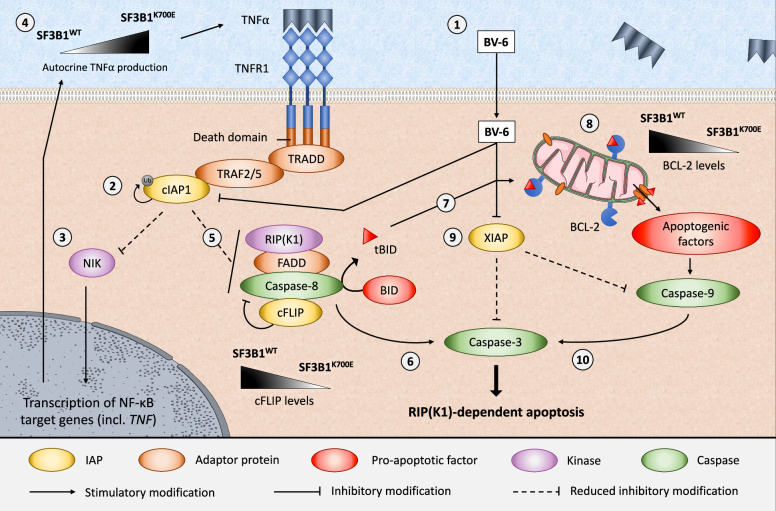
Schematic overview of the susceptibility of *SF3B1*^K700E^-mutated cells to BV-6-induced cytotoxicity, along with the signalling pathways involved. (1) The bivalent SMAC mimetic, BV-6, serves as a dual cIAP1 and XIAP inhibitor. (2) BV-6 causes cIAP1 to auto-ubiquitinate thereby targeting cIAP1 for proteasomal degradation. (3) Loss of cIAP1 promotes non-canonical NF-κB signalling and upregulation of NF-κB target genes including *TNF*. (4) This may promote death receptor signalling through autocrine production of death receptor ligands such as TNF-α (5) Disruption of RIP(K1) ubiquitination promotes the formation of cytosolic TNF Complex II that (6) activates downstream executioner caspases-3/7. (5) Reduced levels of the endogenous caspase-8 inhibitor, cFLIP, in SF3B1^K700E^ cells further promote caspase-8 activation within TNF Complex II, enabling proteolytic cleavage of BID. (7) The resulting truncated BID (tBID) can then be bound and sequestered by pro-survival proteins (e.g., BCL-2, BCL-X_L_, and MCL-1), however, when these pro-survival proteins are saturated or absent, (8) as is the case for BCL-2 levels in SF3B1^K700E^ cells, tBID and other pro-apoptotic factors can promote mitochondrial outer membrane permeabilisation (MOMP) and the release of apoptogenic factors. In conjunction with (9) BV-6-mediated inhibition of XIAP, this leads to enhanced activation of the mitochondrial (intrinsic) pathway that, in turn, (10) activates downstream executioner caspases-3/7 and culminates in RIP(K1)-dependent apoptosis. ^*^P < 0.05; ^**^P < 0.01; ***P < 0.001; ^****^P < 0.0001.

Downregulation of both cFLIP_L_ and BCL-2 observed in the mutant SF3B1 models significantly augments activation of executioner caspases in SF3B1^K700E^ cells in response to BV-6. Moreover, it is widely accepted that the ability of SMs to induce apoptosis in vitro is dependent upon autocrine death receptor ligand secretion [33]. The upregulated TNF-α signalling in our SF3B1^K700E^ cells may serve as a potential means to support cell survival and compensate for the imbalance between pro- and anti-apoptotic proteins at baseline. Taken together, the shifted balance of pro- and anti-apoptotic proteins likely primes SF3B1^K700E^ cells for BV-6-induced cell death by facilitating crosstalk between the extrinsic and intrinsic apoptotic pathways (Fig. 6). Importantly, this enhanced sensitivity to BV-6 treatment in cells harbouring the SF3B1^K700E^ mutation provides compelling evidence that patients with cancers carrying this mutation would be good candidates for SM therapy with BV-6 or Birinapant.

## Methods

### Cell lines and culture

The SF3B1 isogenic model was generated and maintained as previously described ^11^. NCI-H2591 (RRID:CVCL_A543) (*SF3B1*^WT^) and NCI-H2595 (RRID:CVCL_A545) (*SF3B1*^K700E^) cells were obtained from Cosmic-CLP and maintained as per the supplier’s guidance. Polyclonal, stable K-562 cells overexpressing the long and short splice variants of c-FLIP*, and BCL-2** were generated by retroviral and lentiviral transductions, respectively, followed by selection with 10 μg/mL puromycin (ant-pr-1; InvivoGen). All cell line stocks have been authenticated by short tandem repeat profiling and were regularly verified as *Mycoplasma*-free.

### Plasmids

*pBABE-puro plasmids (RRID:Addgene_1764) [35] containing the long and short isoforms of c-FLIP were gifts from Daniel Longley [36, 37].

**pCDH-puro-Bcl2 was a gift from Jialiang Wang (RRID:Addgene_46971) [38].

### Drug screening

SF3B1^WT^ and SF3B1^K700E^ K-562 cells were screened, and *Z*-score values were calculated as previously described [39, 40].

### Compounds

BV-6 (S7597), Xevinapant (S2754), Ferrostatin-1 (S7243), Necrostatin-1 (S8037), and z-VAD-FMK (S7023) were all purchased from Selleckchem. Birinapant (TL32711) (A4219-APE) was purchased from APExBio. Necrosulfonamide (S8251; Selleckchem) was a gift from Richard Turkington. MG-132 (J63250.MA) was purchased from Thermo Fisher Scientific.

### Cell viability assay

Cell viability was assessed using the CellTiter-Glo 2.0 luminescent assay (G9242; Promega) following the manufacturer's guidelines, and luminescence was measured using a BioTek Synergy2 plate reader (BioTek).

### Caspase-Glo assay

Protease activity levels for caspases-3/7, -8, and -9 were assayed using the Promega Caspase-Glo 3/7 (G8090), Caspase-Glo 8 (G8200), and Caspase-Glo 9 (G8210) assay systems according to the manufacturer’s instructions.

### Flow cytometry

Samples were analysed on a BD Accuri C6 Plus flow cytometer and BD CSampler Plus software (version 1.0.23.1).

#### TNFR1 cell surface staining

SF3B1^WT^ and SF3B1^K700E^ cells were collected, counted, and diluted to 10^7^ cells/mL with eBioscience Flow Cytometry Staining Buffer (#FAB225G; Bio-Techne). Samples were Fc-blocked with 5 μL of Human TruStain FcX (422302; BioLegend) for 15-min at room temperature (RT). A 10 μL volume of AF488-conjugated TNFR1 antibody (Ex: 488 nm, Em: 533/30 nm) was added and incubated for 30-min at RT in the dark. Cells were resuspended in 0.5 mL of eBioscience Flow Cytometry Staining Buffer following washing. Five microliters of 7-AAD viability dye (#420404; Thermo Fisher Scientific) were added to each sample, followed by a 5-minute incubation at RT in the dark prior to data acquisition. All experiments were carried out with an IgG1 isotype control antibody (#IC002G; Bio-Techne).

#### Annexin V/7-AAD staining

Cell surface phosphatidylserine expression was assessed using the FITC Annexin V Apoptosis Detection Kit with 7-AAD (Cat #640922), purchased from BioLegend, according to their guidelines.

### Enzyme-linked immunosorbent assay (ELISA)

TNFα levels in cell culture supernatants were quantified using the ultrasensitive Human TNF alpha ELISA (#KHC3013; Thermo Fisher Scientific). Samples were run neat and assayed as per the manufacturer’s instructions.

### Immunoprecipitation

Cells were lysed in CHAPS buffer (30 mM Tris pH 7.5, 150 mM NaCl, 1% CHAPS). Two micrograms of caspase-8 antibody (#ALX-804-242; Enzo) were conjugated to 25 μL Pierce Protein A/G Magnetic beads (88802; Thermo Fisher Scientific). One milligram of protein lysate was immunoprecipitated overnight at 4 °C. Mouse IgG1 isotype control (X093101; Dako) was purchased from Agilent. Co-immunoprecipitation experiments were analysed by western blotting.

### Western blotting

Whole-cell extracts (WCE) were prepared by lysing cells in two volumes of erythrocyte lysis buffer (ELB) (250 mmol/L NaCl, 5 mmol/L EDTA, 50 mmol/L HEPES, 0.1% v/v NP-40; pH 7.5) supplemented with 1X Halt protease and phosphatase inhibitor cocktail (78440; Thermo Fisher Scientific). Thirty to 60 μg of WCE was resolved on 4–12% Bolt Gels (Thermo Fisher Scientific). Protein was transferred to nitrocellulose membranes (Thermo Fisher Scientific) and blotted using the following antibodies: NF-κB2 p52/p100 (sc-7386), BCL-2 (sc-509), and β-actin (sc-47778) from Santa Cruz Biotechnology; Caspase-8 (ALX-804-242-C100, Enzo); cIAP1 (10022-1-AP, Proteintech); BCL-X_L_ (2762), FADD (2782), MCL-1 (5453), XIAP (2045), FLIP (56343), and RIP (3493) from cell signalling technology (CST). Anti-mouse (7076) and anti-rabbit (7074) HRP-conjugated secondary antibodies were also purchased from CST.

### RNA-seq, gene set enrichment analysis (GSEA) and differential splicing

RNA-seq libraries were prepared using RNeasy Plus Mini Kits (741434; QIAGEN). Sequencing was performed in the Genomics Core Technology Unit at Queen’s University Belfast. Reads were aligned using STAR [41], and transcripts were quantified using DESeq2 [42]. EventPointer [43] was used to assess aberrant splicing events between SF3B1^K700E^ and WT controls.

GSEA was performed using the GSEA v4.1.0 software (www.broadinstitute.org/gsea/). Cytoplasmic RNA preparations, sequencing, and downstream data analysis, including differential splicing analysis, using dSpliceType, were carried out as described previously [11].

### Quantitative PCR

cDNA was synthesised using the High-Capacity cDNA Reverse Transcription Kit (#4368814; Thermo Fisher Scientific) according to the manufacturer’s instructions. qPCR was carried out using FastStart Universal SYBR Green Master (Rox) mix (#4913914001; Merck Life Science UK Ltd), QIAGEN QuantiTect Primer Assays***, and the LC480 thermal light cycler (Roche) according to the manufacturer’s instructions.

***Hs_BCL2_1_SG (QT00025011); Hs_BCL2L1_1_SG (QT00236712); Hs_CFLAR_1_SG (QT00064554); Hs_MCL1_1_SG (QT00094122)

### Statistical analysis

GraphPad Prism (version 9.1.2) was used to determine all statistical comparisons. One-way and two-way ANOVA tests were performed with a multiple comparisons post-test as indicated. A *P*-value of 0.05 or less was considered statistically significant. Except for the apoptosis-inducing agent drug screen and MI-2 72 h dose-responses, means were compared across a minimum of three biological replicates, with at least two technical replicates averaged where appropriate.

## Supplementary information

Supplementary data

Supplementary Table 1

Supplementary Table 2

Uncropped Blots

## Data Availability

RNA-seq fastq files are available on the BioProject database using ID: PRJNA1065630.
