# Supplementary material for: The cancer-associated SF3B1K700E spliceosome mutation confers enhanced sensitivity to BV-6-induced cytotoxicity
Source: Cell Death Dis. 2025 Jul 1;16(1):476. doi: 10.1038/s41419-025-07790-y (PMC12216663; doi:10.1038/s41419-025-07790-y)
Supplement: Supplementary file 1 — Supplementary data [file 41419_2025_7790_MOESM1_ESM.pdf]

Supplementary Figure 1

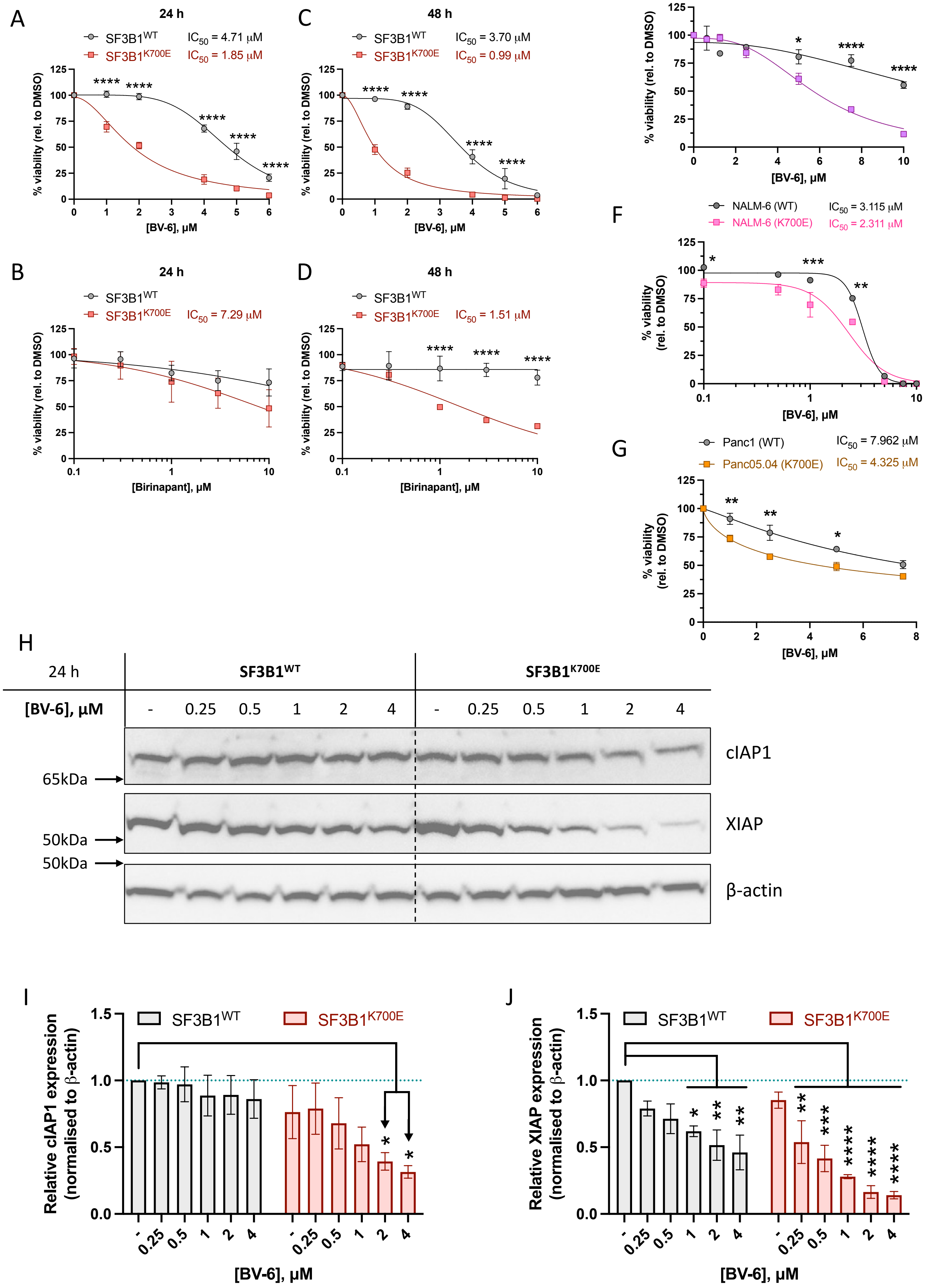

Supplementary Figure 1: Assessment of cell viability following 24- (A-B), 48- (C-D) or 72- (E-G) hour treatment of SF3B1^{WT} and SF3B1^{K700E} K-562 (A-D), H-2591/H-2595 (E), NALM-6 (F) or Panc1/Panc05.04 (G) cells with increasing concentrations of BV-6 or Birinapant (Mean ± SEM; n = 3-5; 2-way ANOVA with Šidák's multiple comparisons). (H) Representative Western blot of two inhibitor of apoptosis proteins from SF3B1^{WT} and SF3B1^{K700E} cells following a 24 h treatment with the indicated BV-6 concentrations. (I-J) Blot densitometry quantified cIAP (I) and XIAP (J) protein expression (Mean ± SEM; n = 3; 2-way ANOVA with Dunnett's multiple comparisons). *, $P < 0.05$; **, $P < 0.01$; ***, $P < 0.001$; ****, $P < 0.0001$.

Supplementary Figure 2

A

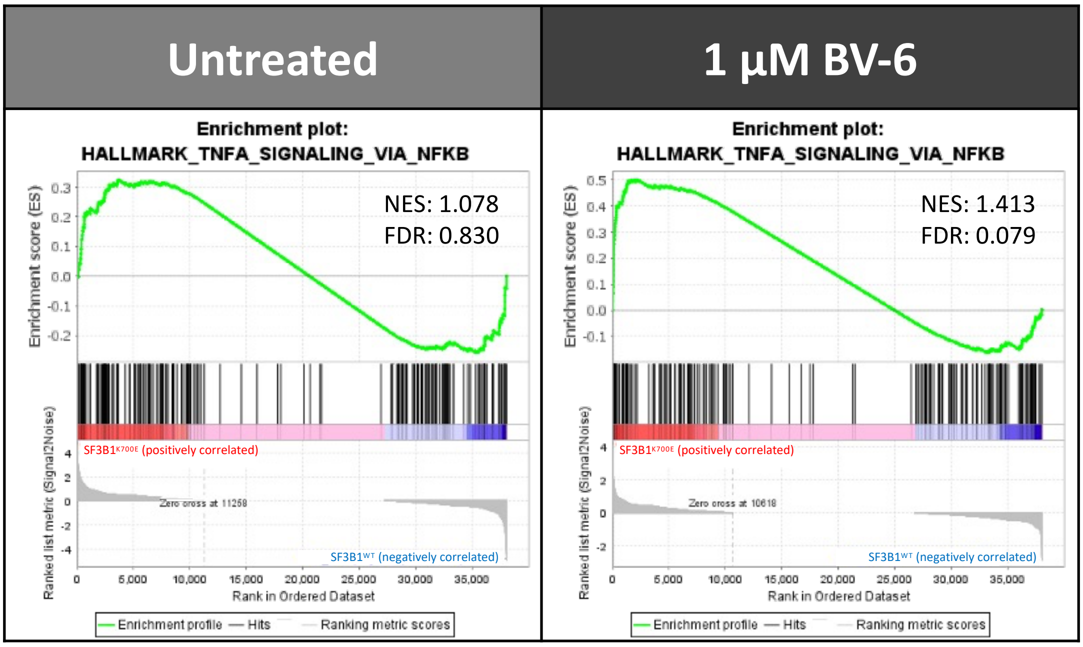

B

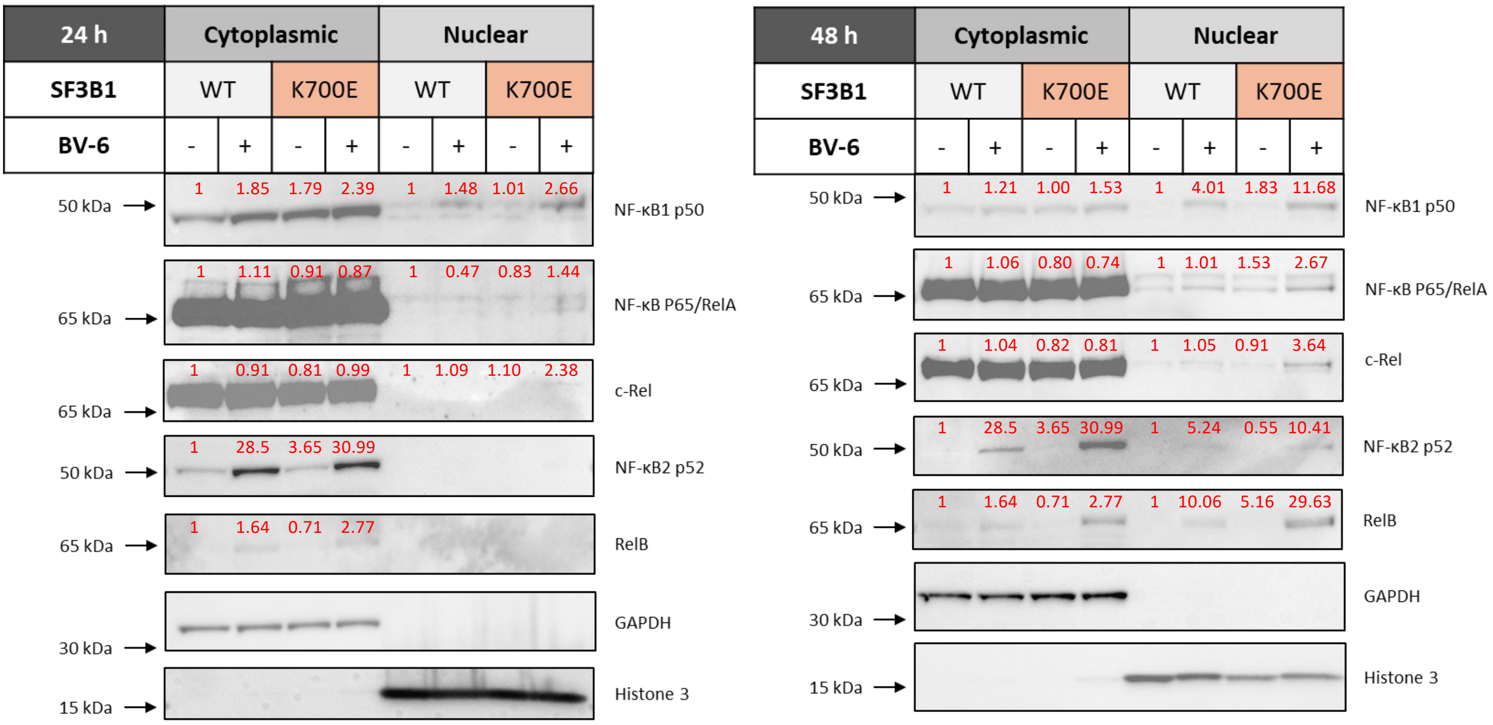

Supplementary Figure 2:

(A) Gene set enrichment analysis (GSEA) plots showing enrichment of the 'TNFα signalling via NF-κB' Hallmark in SF3B1^{K700E} cells compared to WT control in untreated and BV-6 treated cells (n = 3). NES, Normalised Enrichment Score; FDR, False Discovery Rate. (B) Representative Western blot of NF-κB1 and NF-κB2 signalling proteins from SF3B1^{WT} and SF3B1^{K700E} K-562 cells following a 24 h treatment with the indicated concentrations of BV-6. Mean ± SEM; (24h n=2), (48h n = 3); Blot densitometry data is shown normalized to GAPDH (Cytoplasmic fraction) or Histone H3 (nuclear fraction) relative to WT control for each fraction.

Supplementary Figure 3

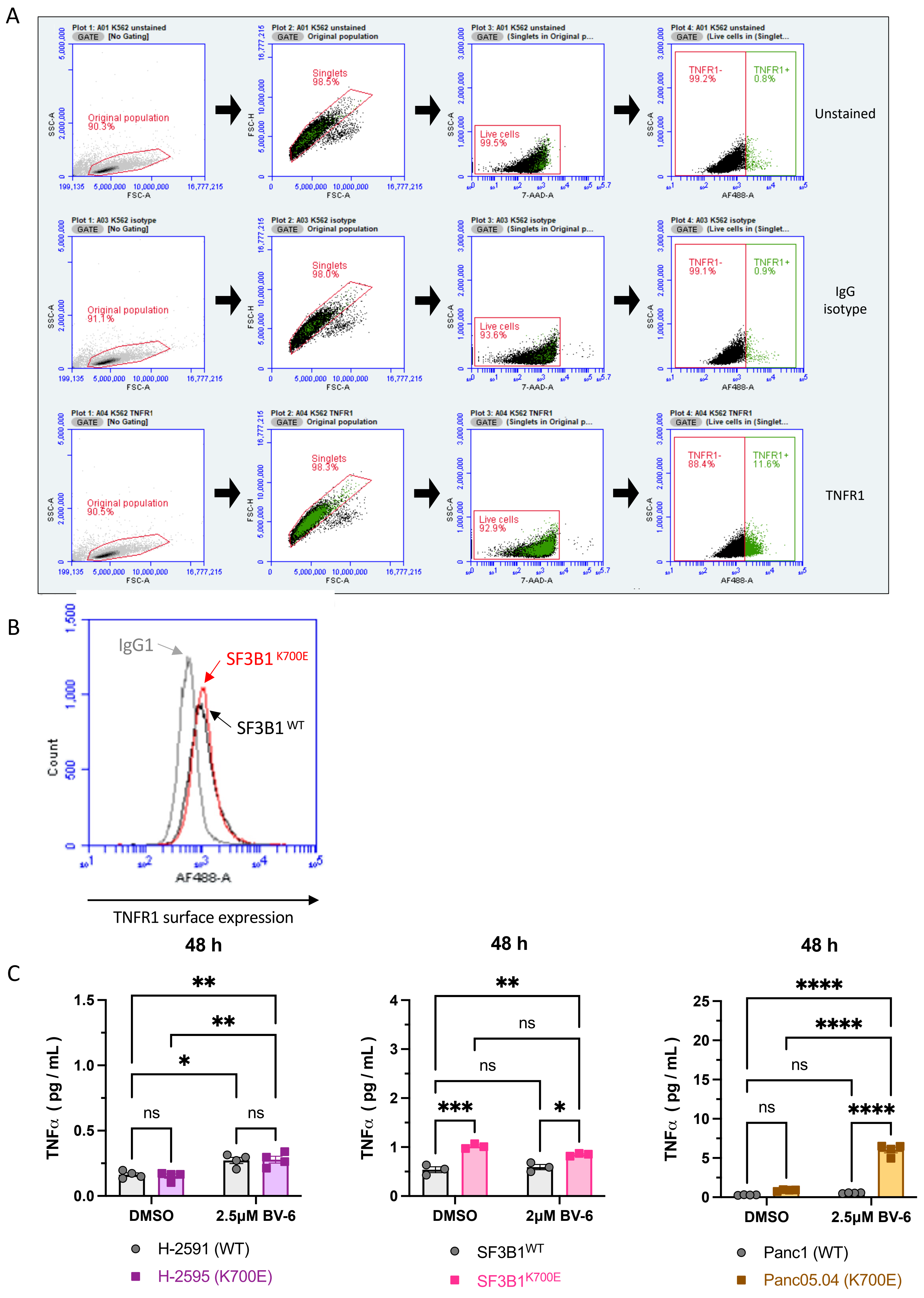

Supplementary Figure 3:

(A) Flow cytometry gating strategy for characterisation of TNFR1 surface expression. (B) Representative histogram illustrating the counts and mean fluorescence intensities (MFIs) of the AF488-conjugated TNFR1 antibody binding to *SF3B1*^{WT} (MFI = 1,176) and *SF3B1*^{K700E} (MFI = 1,225) cells, compared with an IgG1 isotype control (MFI = 612). (Mean ± SEM; n = 3; Mann-Whitney test). *, P < 0.05; **, P < 0.01; ***, P < 0.001; ****, P < 0.0001; NS, non-significant. (C) Autocrine (soluble) TNFα levels within the cell culture media of SF3B1^{WT} and SF3B1^{K700E} H2591/H2595, NALM-6 and Panc1/Panc05.04 cells, following 48-h treatment with 0.1 % DMSO vehicle or 1 μM BV-6 (Mean ± SEM; n = 3; 2-way ANOVA with Tukey's multiple comparisons).

Supplementary Figure 4

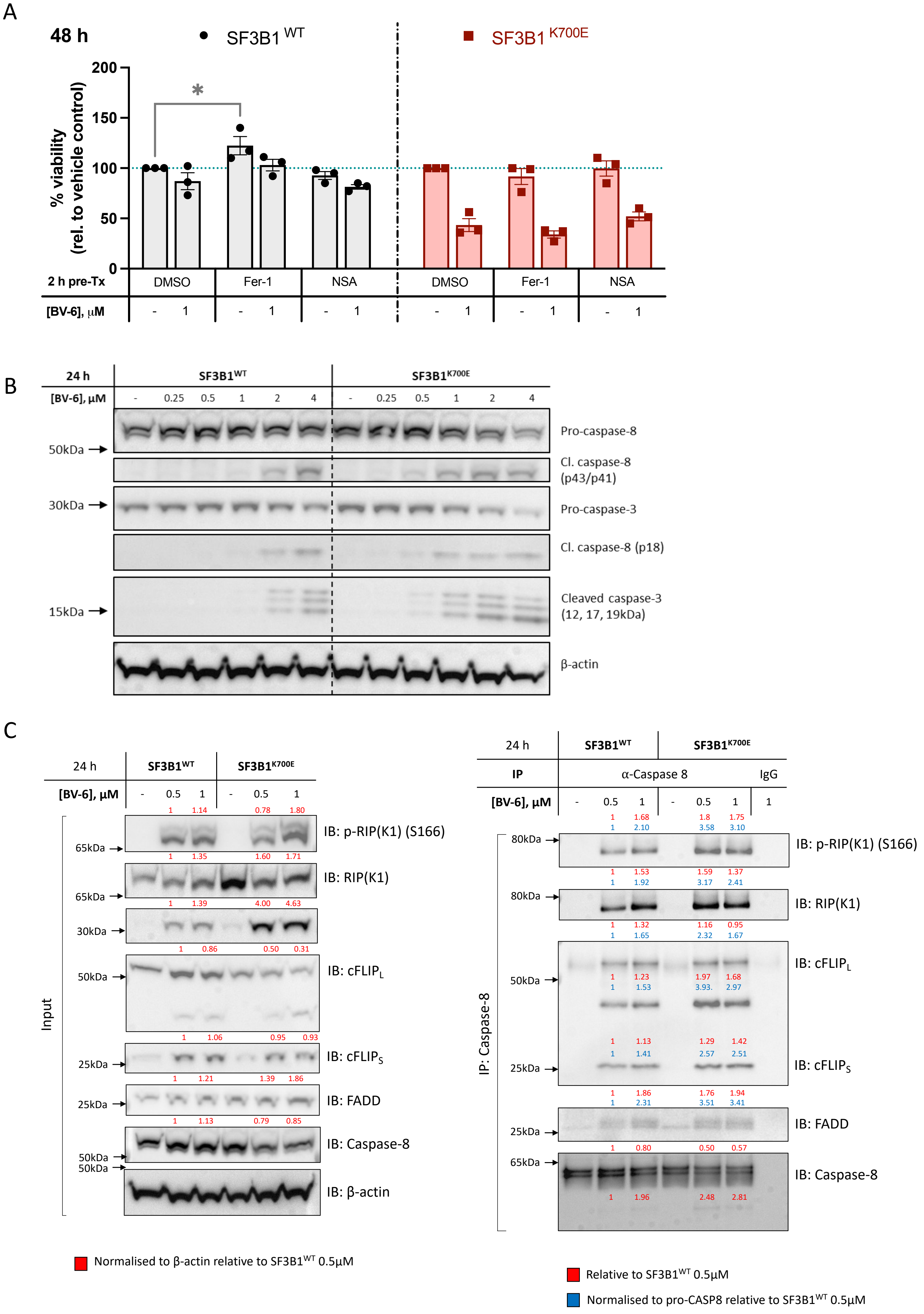

Supplementary Figure 4:

(A) Viability assessment of $SF3B1^{WT}$ and $SF3B1^{K700E}$ cells pre-treated for 2 h with 0.1 % DMSO vehicle control, 1 µM Ferrostatin-1, or 1 µM Necrosulfonamide, followed by a 48-hour incubation with 0.2 % DMSO vehicle or 1 µM BV-6 (Mean ± SEM; n = 3; 2-way ANOVA with Dunnett's multiple comparisons). B) Representative Western blot analysis of total and cleaved caspase8 and 3 proteins in $SF3B1^{WT}$ and $SF3B1^{K700E}$ cells following treatment with the indicated concentrations of BV-6 for 24h. C) Relative blot densitometry of figure 3D.

Supplementary Figure 5

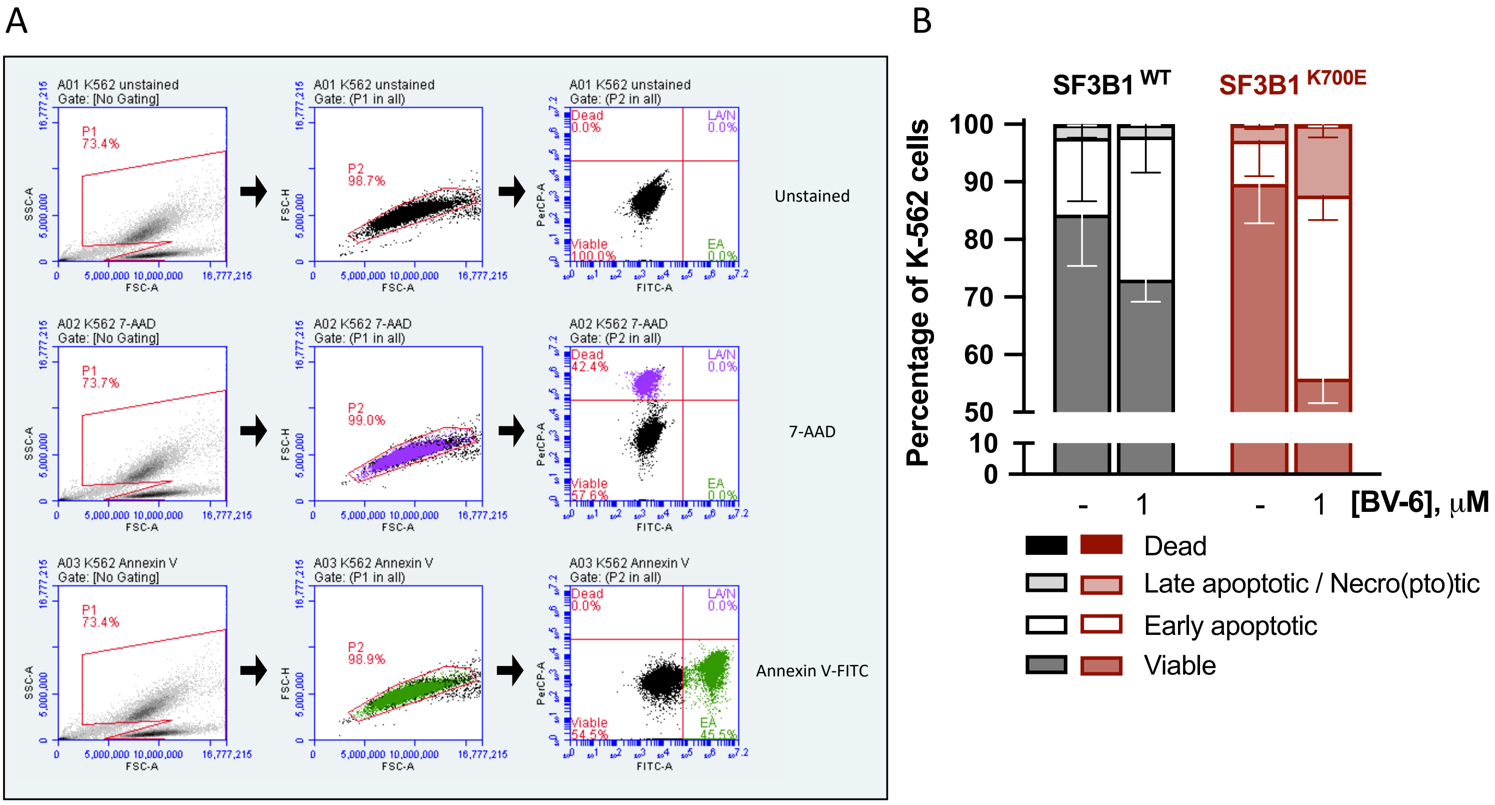

Supplementary Figure 5:

(A) Flow cytometry gating strategy for assessment of the proportion of apoptotic K-562 cells, depicting the acquired data for (half heat-killed) unstained cells and the two single stain controls: 7-AAD viability dye and FITC-conjugated Annexin V. EA: early apoptotic (Annexin V-positive, 7-AAD-negative); LA/N: late apoptotic / necro(pto)tic (Annexin V-positive, 7-AAD-positive). (B) Stacked bar chart depicting the various proportions of dead, late apoptotic/necro(pto)tic, early apoptotic, and viable cells following a 48 h incubation with 0.1 % DMSO vehicle or 1 μM BV-6 (Mean ± SEM; n = 3-4). *, $P < 0.05$; **, $P < 0.01$; ***, $P < 0.001$; ****, $P < 0.0001$.

Supplementary Figure 6

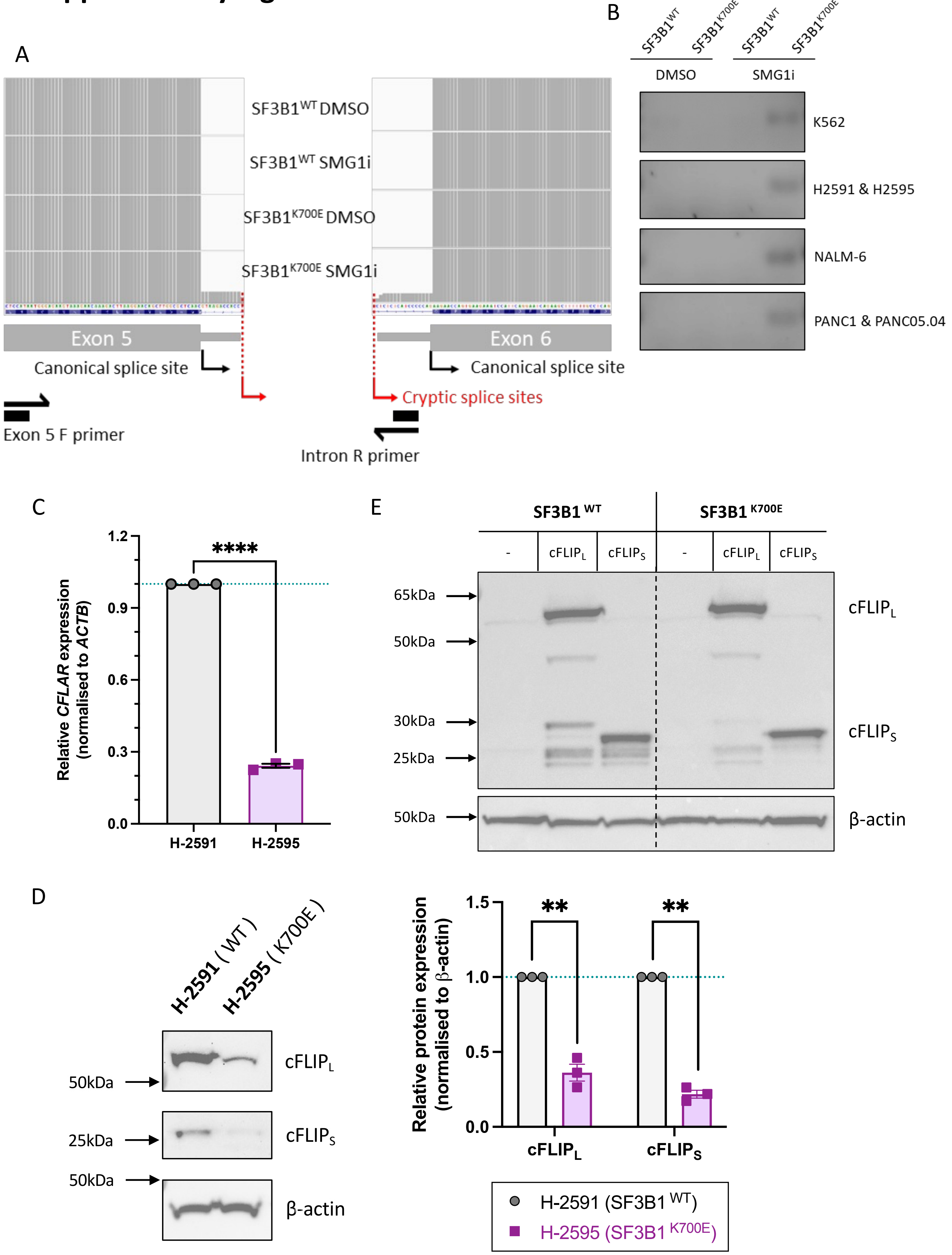

Supplementary Figure 6: (A) Visualization of RNA-Seq traces for the CFLAR gene (exon5-intron6- and intron6-exon6 junctions) in SF3B1^{WT} and SF3B1^{K700E} K-562 cells following treatment with vehicle and SMG1 inhibitor, using Integrative Genomics Viewer (IGV). Cryptic splice sites are observed in the *CFLAR* gene within intron 6, in the SF3B1-mutant cell line following inhibition of NMD. (B) RT-PCR confirmation of *CFLAR* intron 6 cryptic splice site usage in SF3B1-mutant cells showing amplification of the cryptic intronic sequence. (C) qRT-PCR analysis of *CFLAR* transcript levels in H-2591 (SF3B1^{WT}) and H-2595 (SF3B1^{K700E}) lung mesothelioma cells (Mean ± SEM; n = 3; Unpaired t test). (D) Representative Western blot for the long (cFLIP$_L$) and short (cFLIP$_S$) isoform protein levels of cFLIP in H-2591 (SF3B1^{WT}) and H-2595 (SF3B1^{K700E}) lung mesothelioma cells at baseline, along with associated densitometry data (Mean ± SEM; n = 3; Multiple unpaired t tests with Welch's correction). (E) Western blot validation of cFLIP$_L$ or cFLIP$_S$ overexpression in SF3B1^{WT} and SF3B1^{K700E} K-562 cells. *, P < 0.05; **, P < 0.01; ***, P < 0.001; ****, P < 0.0001.

Supplementary Figure 7

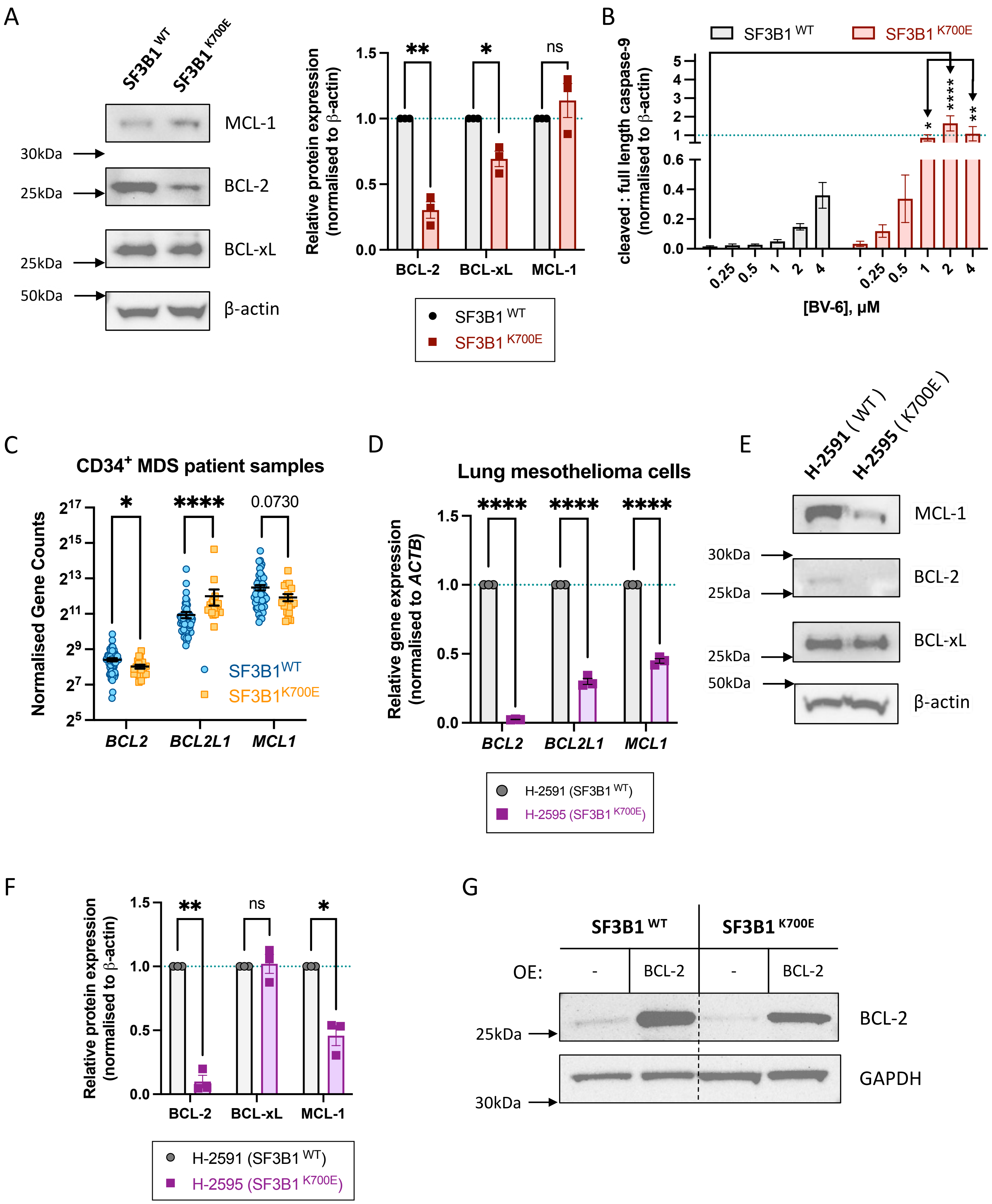

Supplementary Figure 7: (A) Representative western blot of MCL-1, BCL-2, and BCL-xL protein levels in SF3B1^{WT} and SF3B1^{K700E} cells at baseline, along with associated densitometry data (Mean ± SEM; n = 3; Multiple unpaired t tests with Welch's correction). (B) Densitometry data associated with Figure 5D, for the ratios of cleaved to full length caspase-9 (Mean ± SEM; n = 3; 2-way ANOVA with Dunnett's multiple comparisons). (C) Normalised counts of pro-survival *BCL2*, *BCL2L1*, and *MCL1* genes from SF3B1^{WT} and SF3B1^{K700E} CD34-enriched MDS patient samples (Mean ± SEM; n = 19-54; Multiple Mann-Whitney tests). (D) qRT-PCR analysis of the transcriptomic data for *BCL2*, *BCL2L1*, and *MCL1* genes from H-2591 (SF3B1^{WT}) and H-2595 (SF3B1^{K700E}) lung mesothelioma cells (Mean ± SEM; n = 3; Multiple unpaired t tests with Welch's correction). (E) Representative Western blot of MCL-1, BCL-2, and BCL-xL protein levels in H-2591 (SF3B1^{WT}) and H-2595 (SF3B1^{K700E}) lung mesothelioma cells at baseline. F) Blot densitometry quantification of MCL-1, BCL-2, and BCL-xL protein levels in H2592 and H2595 cells (Mean ± SEM; n = 3; Multiple unpaired t tests with Welch's correction). (G) Western blot validation of BCL-2 overexpression in SF3B1^{WT} and SF3B1^{K700E} K-562 cells. *, P < 0.05; **, P < 0.01; ***, P < 0.001; ****, P < 0.0001; NS, non-significant.
